# Differences in monocyte subsets are associated with short‐term survival in patients with septic shock

**DOI:** 10.1111/jcmm.15791

**Published:** 2020-09-19

**Authors:** Marcela Hortová‐Kohoutková, Petra Lázničková, Kamila Bendíčková, Marco De Zuani, Ivana Andrejčinová, Veronika Tomášková, Pavel Suk, Vladimír Šrámek, Martin Helán, Jan Frič

**Affiliations:** ^1^ International Clinical Research Center St. Anne's University Hospital Brno Brno Czech Republic; ^2^ Department of Biology Faculty of Medicine Masaryk University Brno Czech Republic; ^3^ Department of Anesthesiology and Intensive Care Faculty of Medicine Masaryk University Brno Czech Republic; ^4^ Institute of Hematology and Blood Transfusion Prague Czech Republic

**Keywords:** cytokines, immunity, inflammation, monocytes, sepsis, septic shock, T cells

## Abstract

Sepsis is characterized by dynamic changes of the immune system resulting in deregulated inflammation and failure of homoeostasis and can escalate to septic shock. Circulating monocytes and other innate immune cells are among the first ones to recognize and clear pathogens. Monocytes have an important role in sepsis and septic shock and have been studied as potential diagnostic markers. In total, forty‐two patients with septic shock were recruited and blood samples obtained within first 12 hours of ICU admission. We showed that frequency of classical and intermediate monocytes assessed at the time of admission to the intensive care unit are significantly distinct in patients with septic shock who survived longer that five days from those who died. These parameters correlate significantly with differences in serum levels of inflammatory cytokines MCP‐1, IL‐6, IL‐8, IL‐10, and IL‐18, and with the proportion of helper and cytotoxic T cells. The described changes in frequency of monocyte subsets and their activation status may predict short‐term septic shock survival and help with fast identification of the group of vulnerable patients, who may profit from tailored therapy.

## 1 INTRODUCTION

Sepsis is defined as the uncontrolled response of the host immune system to infection leading to subsequent organ dysfunction^1^; it is a life‐threatening syndrome with increasing incidence worldwide. Sepsis can escalate to septic shock, representing a clinical condition of a persisting low blood pressure with vasopressor requirement and volume resuscitation to maintain a sufficient mean blood pressure with current high levels of blood lactate. In‐hospital mortality ranges from 10% for sepsis, to 39% for septic shock.^1^, ^2^ Although sepsis is a multifactorial disorder, regaining control of the inflammatory response seems to be crucial to maintain homoeostasis but despite decades of effort, mortality remains high.^3^ Clinical management of sepsis relies on early recognition of the condition, followed by identification and control of the source of sepsis, with simultaneous administration of antimicrobials and organ support to give patients time to recover.^1^, ^4^, ^5^ Early identification of patients with sepsis is based on evaluation of clinical characteristics and laboratory markers equating to a change in the sequential organ failure assessment (SOFA) score of 2 points, or more.^1^ Many predictors of poor clinical outcome—such as high SOFA score, refractory course of shock, persistent hyperlactatemia, and hypothermia—have been described.^6^ However, no single, standardized predictive marker currently exists to define the group of patients with the worst prognosis.

Monocytes carry the long‐term burden of sepsis.^7^, ^8^ These cells are activated by pattern recognition receptors (PRRs) and sepsis‐associated hypoxia^9^, ^10^; the hypoxia‐inducible factor (HIF‐1α) has been identified as having a key role in the functional reprogramming of these cells.^7^ Monocytes can be categorized into three major subpopulations: classical (CD14^+^ CD16^−^), intermediate (CD14^+^ CD16^+^), and non‐classical (CD14^lo^ CD16^+^) monocytes.^11^ Fingerle et al reported that CD16^+^ cells can represent up to 50% of all monocytes during sepsis.^12^ This finding led to studies of functional changes in monocytes during sepsis, although correlations between monocyte counts and sepsis severity have only been reported in the past 2 years.^13^, ^14^, ^15^ In the search for diagnostic markers of sepsis progression, promising results have been provided by phenotypic changes in monocytes and the presence of monocyte‐produced molecules in plasma.^16^ However, studies on the role of monocytes during sepsis have not generally considered the complexity of monocyte biology.^15^ The individual mechanistic roles of the three distinct monocyte subsets in sepsis progression remain to be investigated. Moreover, monocyte role in prediction of short‐term survival in patients with sepsis has not been studied.

Sepsis affects most of the immune system functions, profound changes indeed impact leucocyte subsets including CD4^+^ T cells.^17^ CD4^+^ T cells are important for regulation and orchestration of immune response and their impairment leads to loss of functional immune cells and subsequent immunosuppression; characteristic feature very often induced during sepsis.^18^, ^19^

In this study, we address whether the differences in monocyte subsets observed in patients with septic shock at the time of admission correlate with the severity of septic shock progression, including cytokine expression, changes in SOFA score, and early mortality. The diagnosis within the early stages of septic shock is an important factor in patient prognosis.

## 2 MATERIAL AND METHODS

### 2.1 Cohort design

In this prospective, observational cohort study, adult patients with early septic shock who were admitted to the intensive care unit (ICU) at St. Anne's University Hospital in Brno, Czech Republic were consecutively enrolled. Patients with chronic immunosuppression and those who had received antibiotic therapy for more than 2 days were excluded. All patients were treated with tailored therapy according to current guidelines.^20^ The cohort was divided for study purposes into two groups: those who survived longer than five days after admission and those who died within five days (early deceased). Written informed consents were obtained from all enrolled patients and all procedures and protocols were approved by the institutional ethic committee (4G/2018).

### 2.2 Blood sample isolation and preparation

Blood samples were obtained from patients within 12 hours of admission to the ICU and were processed within 2 hours of collection. Peripheral blood mononuclear cells (PBMCs) were isolated from blood by gradient centrifugation using Lymphoprep^®^ (Alere Technologies AS; Oslo, Norway) (density 1.077 g/mL) following the manufacturer's recommendations. Serum was collected in vials facilitating coagulation, centrifuged and immediately frozen and stored at −80°C.

### 2.3 Immunophenotyping of samples

To obtain the phenotype of monocytes, PBMCs were labelled using lineage antibodies {CD66b‐biotin (RRID:AB_2566608) (BioLegend, Inc, San Diego, CA. USA), anti‐CD3‐biotin (RRID:AB_466323), anti‐CD19‐biotin (RRID:AB_466388), anti‐CD20‐biotin (RRID:AB_657690), anti‐CD56‐biotin (RRID:AB_10596499), anti‐CD235α‐biotin (RRID:AB_494036), followed by streptavidin eFluor450 (RRID:AB_10359737)}, anti‐CD45‐PerCP‐eFluor710 (RRID:AB_11041112), anti‐CD16‐APC (RRID:AB_2016663), anti‐CD86‐PE (RRID:AB_10732345) anti‐HLA‐DR‐FITC (RRID:AB_2572542) (Affymetrix, Inc, Santa Clara, CA, USA), and anti‐CD14‐BV510 (RRID:AB_2833091) (Sony Biotechnology, Inc, San Jose, CA, USA). A LIVE/DEAD fixable near‐IR dead cell stain kit (Thermo Fisher Scientific, Inc, Waltham, MA USA) was used to exclude dead cells from analysis. Monocyte subsets were classified as classical (CD14^+^ CD16^−^), intermediate (CD14^+^ CD16^+^), and non‐classical (CD14^lo^ CD16^+^).

The T cell immunophenotype was determined by labelling with anti‐CD3‐APC‐Cy7 (RRID:AB_2563410), anti‐CD8‐FITC (RRID:AB_314124), (BioLegend, Inc), and anti‐CD4‐BV510 (RRID:AB_2744424) (BD Biosciences, Franklin Lakes, NJ, USA). CD3^+^ T cells were used to distinguish between helper (T_h_) CD4^+^ and cytotoxic (T_c_) CD8^+^ T cells. Sample acquisition was performed using FACSCanto^®^ (BD Biosciences) and data were analysed using FlowJo^®^ software (FlowJo, LLC Ltd, Ashland, OR, USA).

### 2.4 Cytokine detection

Preparation and measurement of samples were performed using a LEGENDplex™ kit (BioLegend, Inc) according to the manufacturer's guidelines, with overnight incubation of twice‐diluted serum samples with beads specific for following proteins: monocyte chemoattractant protein (MCP)‐1, interleukin (IL)‐6, IL‐8, IL‐10, IL‐18, and IL‐33. Acquisition of samples was done on FACS Canto (BD Biosciences) and data were analysed using LEGENDplex™ software.

### 2.5 Statistical analysis

Prism^®^ (GraphPad Software, LLC, Ltd, La Jolla, CA, USA) software was used for statistical analysis. Data were tested for normal distribution and statistical tests were applied as appropriate. Error bars are represented by SD. Statistical tests used are specified in the figure legends. The level of statistical significance was determined: *(*P* < 0.05), **(*P* < 0.01), and ***(*P* < 0.001). Correlation analysis was performed using Spearman's rank correlation coefficient and visualization was done in R studio by corrplot package. AUC of ROC curves were calculated using Prism^®^ software.

## 3 RESULTS

### 3.1 Demographic and clinical characteristics of patients

We enrolled a total of 41 patients (all Caucasians), 33 of whom survived longer than five days after admission. The characteristics of the cohort as a whole and by group are shown in Table 1, Figure S1. The mean age of patients was 71.3 years (range 49‐89 years) and mean SOFA score of enrolled patients was 11.46 indicating septic shock. The most frequent comorbidities include ischaemic heart disease, obesity, ischaemic disease of the lower limbs, obesity and diabetes mellitus. The most common cause of septic shock in the cohort, as a whole was pneumonia (n = 17), where all patients survived past day five. Among those who died before day five, the most common origin of septic shock was infection of the urinary tract. The most frequently detected microbial agents were *Staphylococcus Aureus*, *E Coli*, *Enterococcus faecalis* and *Enterobacter Cloacae*. In 13 patients the primary aetiological agent was not identified.

**TABLE 1:** Demographic and clinical characteristics of patients with septic shock

Characteristic	Total	D5+ Survivors	Early deceased	*P* value
Recruited patients	41	33 (80.5%)	8 (19.5%)	—
Gender				
Female	17 (41.5%)	14 (82.4%)	3 (17.6%)	—
Male	24 (58.5%)	19 (79.2%)	5 (20.8%)	—
Age, mean (range)	71.3 (49‐89)	70.6 (49‐89)	74.1 (66‐85)	.3850
Comorbidities, mean	2.23	2.22	2.25	.8253
BMI, mean	27.80	27.48	29.12	.3699
SOFA, mean	11.46	10.82	14.13	**.0360**
CRP mg/l, mean	224.32	213.38	268.06	.5388
Lactate mmol/l, mean	2.15	1.72	3.90	**.0045**
Origin of septic shock				
Pneumonia	17	17 (100%)	0 (0%)	—
Abdominal infection	7	5 (71.4%)	2 (28.6%)	—
Urosepsis	6	3 (50%)	3 (50%)	—
Soft tissue infection	5	3 (60%)	2 (40%)	—
Mediastinitis	3	3 (100%)	0 (0%)	—
Other	3	1 (33.3%)	2 (66.7%)	—

Abbreviations: BMI, body mass index; CRP, c‐reactive protein; SOFA, sequential organ failure assessment.

### 3.2 Monocyte population characteristics

**FIGURE 1:**
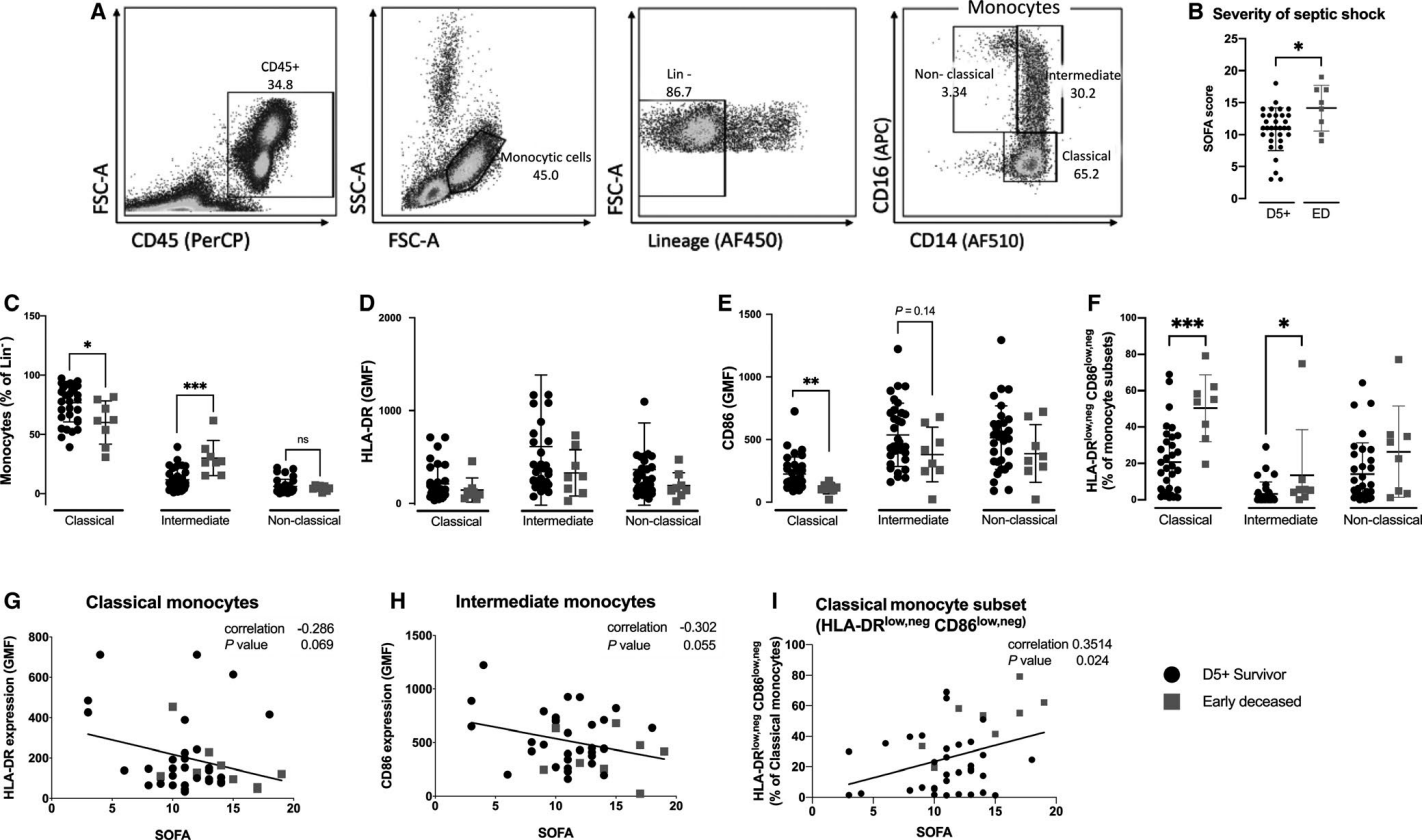
Immunophenotyping of monocytes. A, Gating strategy used for monocyte phenotyping. PBMCs were labelled with CD45 to show all leucocytes, then monocytes were gated from a FSC × SSC plot. Lin^−^ cells (lineage: CD3, CD19, CD20, CD56, CD66b, CD235a) were used to show the distribution of monocyte subsets by CD14 and CD16 labelling. Singlet and live/dead gating strategy plot not shown. B, The differences in septic shock severity, represented by SOFA score of D5+ survivors and early deceased patients. C, Differences in monocyte subsets depending on survival of initial phases of septic shock. There were significant differences in the frequency of classical and intermediate monocytes between D5+ survivors and early deceased patients. D and E, Differences in surface expression of HLA‐DR and CD86 in monocyte subsets. CD86 expression was significantly decreased in the classical monocytes of early deceased patients compared with survivors. F, The presence of HLA‐DR^low,neg^ CD86^low,neg^ cells in monocyte subsets is significantly different in D5+ survivors and early deceased patients. G‐I, Correlations between HLA‐DR and CD86 and sepsis severity (SOFA score) for each monocyte subset. We found the significant correlation between HLA‐DR^low,neg^ CD86^low,neg^ in classical monocyte subset with SOFA score. Data were tested by Mann‐Whitney test, error bars show SD. Correlation statistics (Spearman analysis) were performed using Graphpad software. *(*P* < 0.05), **(*P* < 0.01), ***(*P* < 0.001). FSC, forward scatter; HLA‐DR, human leucocyte antigen‐DR isotype; PBMCs, peripheral blood mononuclear cells; SOFA, sequential organ failure assessment; SSC, side scatter

We analysed monocytes by immunophenotyping their surface markers. CD45^+^ and Lin^−^ (CD3, CD19, CD20, CD56, CD66b, CD235a) cells were subsequently categorized to monocyte subsets based on presence of CD14 and CD16. The gating strategy used for monocyte phenotyping is shown in Figure 1A. We analysed the dependence of patient prognosis on the severity of sepsis (SOFA score) (Figure 1B).

In early deceased patients, the frequency of classical monocytes was significantly decreased (*P* < 0.05) and the frequency of intermediate monocytes was significantly increased (*P* < 0.001), compared to patients who survived past day five (Figure 1C). No differences in non‐classical monocytes were observed. Non‐significant trend towards decreased expression of human leucocyte antigen‐DR isotype (HLA‐DR; an indicator of monocyte activation status) was identified across all monocyte subsets in early deceased patients compared with survivors (Figure 1D). Importantly, the expression of co‐stimulatory molecule CD86 was significantly decreased in the classical monocytes of early deceased patients (*P* < 0.01) and, in the intermediate and non‐classical monocyte subsets, CD86 expression tended to be lower in early deceased patients than in those who survived (Figure 1E). We performed the analysis of HLA‐DR^low,neg^ CD86^low,neg^ cells in all monocyte subsets. Early deceased patients had significantly higher frequency of HLA‐DR^low,neg^ CD86^low,neg^ cells in classical (*P* < 0.001) and intermediate (*P* < 0.05) monocyte subset in comparison to D5+ survivors. Furthermore, the correlation between monocyte activation assessed as surface expression of CD86 and HLA‐DR and SOFA score was evaluated, revealing close to significant negative correlation (*P* = 0.069, *P* = 0.055 respectively; Figure 1G,H). The increased presence of HLA‐DR^low,neg^ CD86^low,neg^ cells in classical monocyte subset revealed significant correlation with sepsis severity represented by SOFA score (*P* = 0.024) (Figure 1I).

### 3.3 Cytokine production and correlation between monocyte characteristics

**FIGURE 2:**
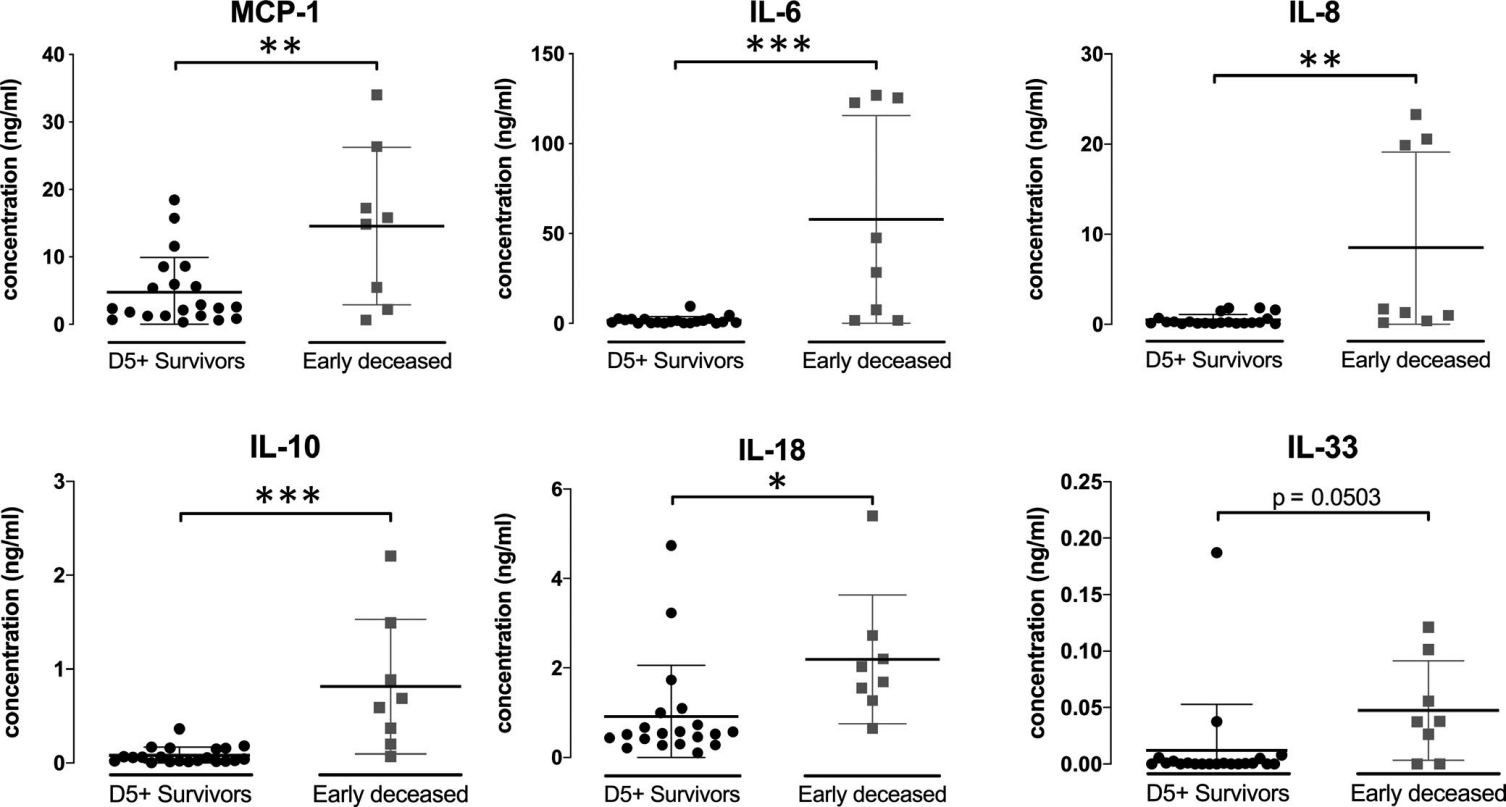
Cytokine production. Significant differences in secreted levels of MCP‐1, IL‐6, IL‐8, IL‐10, and IL‐18 were observed in day 5+ survivor compared with early deceased patients. The difference for IL‐33 was very close to significance (*P* = 0.0503). Data were tested by Mann‐Whitney test, error bars show SD. *(*P* < 0.05), **(*P* < 0.01), ***(*P* < 0.001). IL, interleukin; MCP, monocyte chemoattractant protein

**FIGURE 3:**
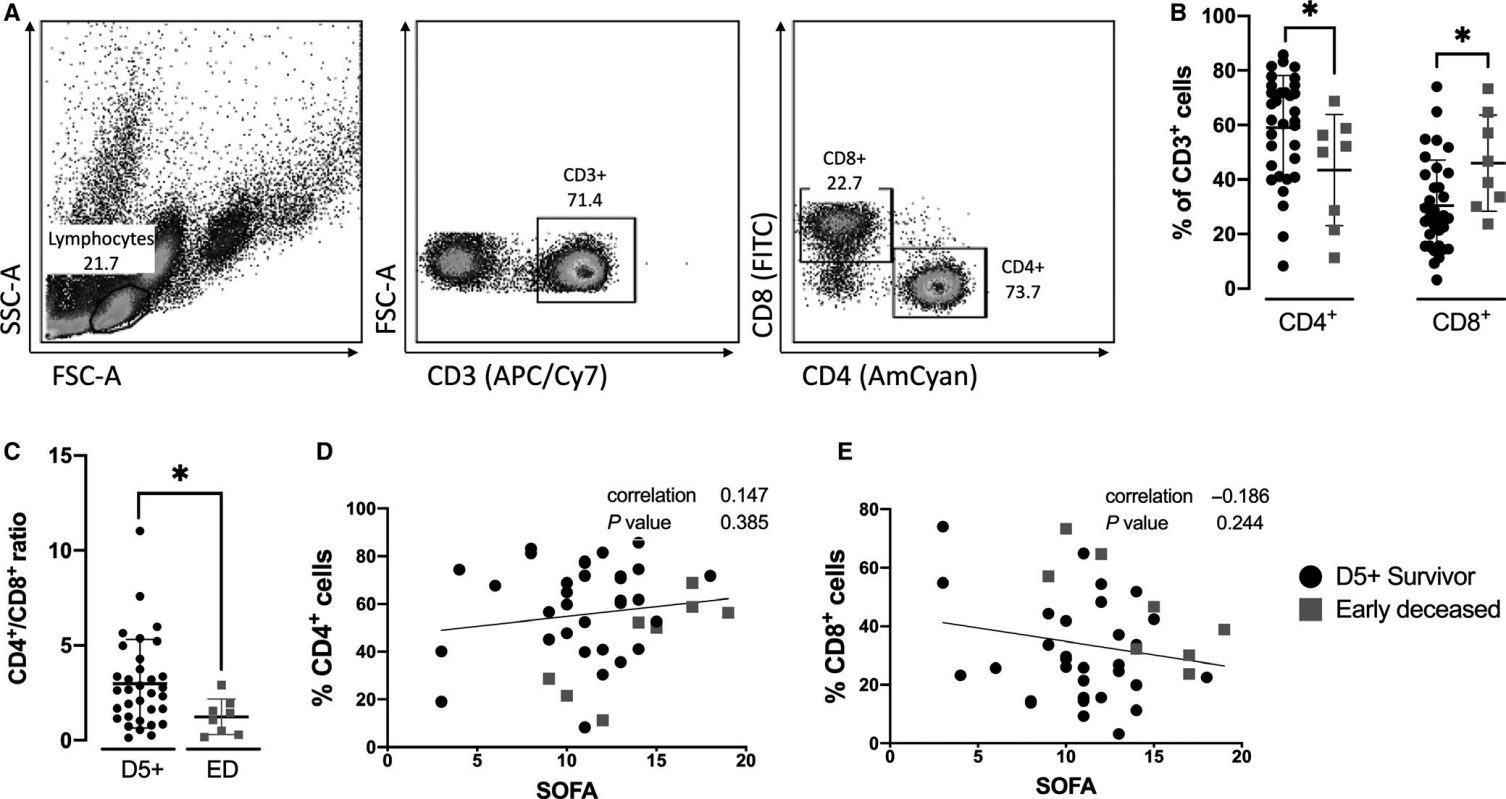
Changes in T cells frequency of patients with septic shock. A, Gating strategy used for T cell phenotyping. Lymphocytes were gated from an FSC x SSC plot. CD3^+^ T cells were used to distinguish CD4^+^ and CD8^+^ T cells. Singlet and live/dead gating strategy plot not shown. B, Differences in CD4^+^ and CD8^+^ cell subsets. There were significantly fewer CD4^+^ cells and significantly more CD8^+^ cells in early deceased patients in day 5+ survivors. C, Ratio of CD4^+^/CD8^+^ cells in D5+ survivors in comparison to early deceased patients. D and E, Correlation between frequency of CD4^+^ and CD8^+^ T cells and sepsis severity (SOFA score). Data were tested by Mann‐Whitney test, error bars are represented by SD. Correlation statistics (Spearman analysis) were performed using Graphpad software. *(*P* < 0.05), **(*P* < 0.01), ***(*P* < 0.001)

**FIGURE 4:**
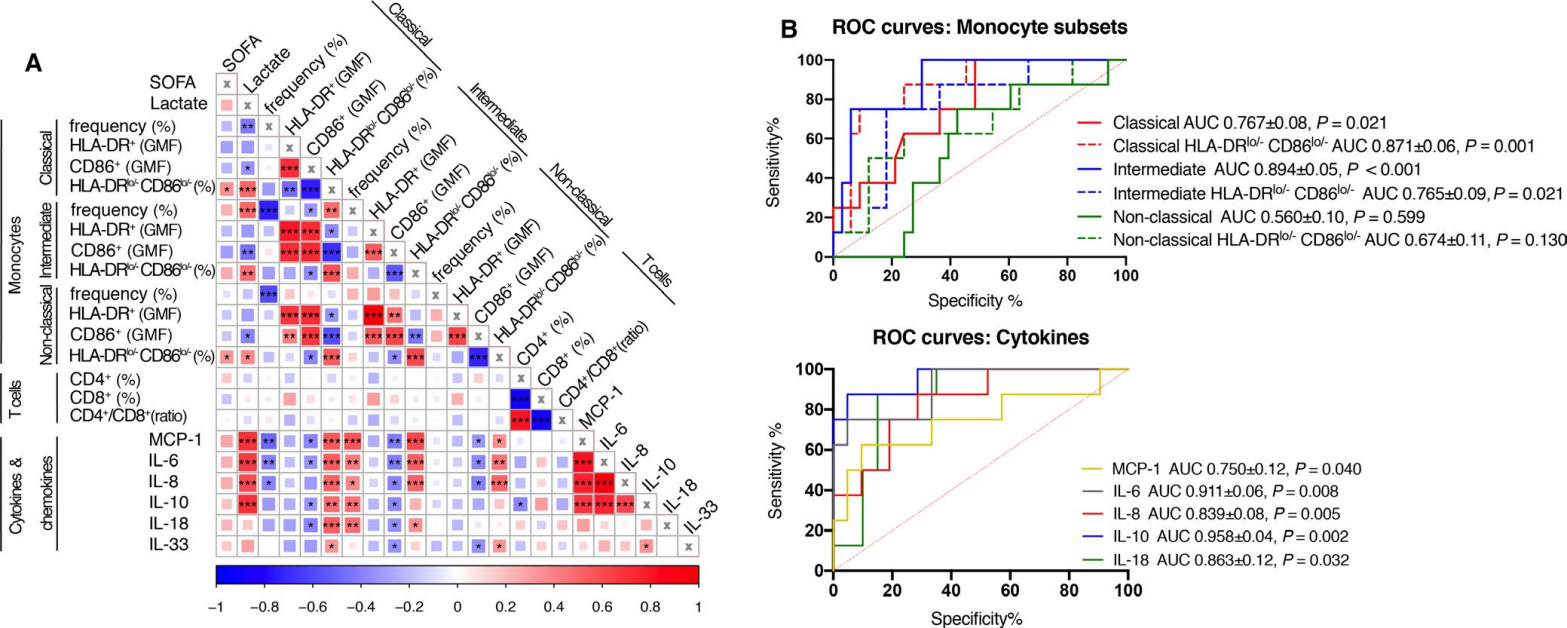
Correlogram of monocytes and T cells’ parameters with produced cytokines. A, Monocyte parameters (Figure 1), T cell parameters (Figure 3) and levels of secreted cytokines (Figure 2) were analysed together. Positive correlations between parameters are displayed in red and negative correlations in blue. The colour intensity and size of the square are proportional to the correlation coefficients. The decreased frequency of classical monocytes strongly negatively correlates with changes of frequency in intermediate and non‐classical monocytes. Importantly, the correlations were found between both activation markers, HLA‐DR and CD86, in all monocyte subsets. Changes in classical monocytes frequency also negatively correlate with MCP‐1, IL‐6 and IL‐8. Contrary to classical monocytes, intermediate monocytes positively correlate with majority of measured cytokines. Important negative correlations were found between CD86 expressed on all monocyte subsets with different cytokines. The reduced frequency of CD4^+^ cells highly negatively correlate with increased frequency of CD8^+^ cells (*P* < 0.001). Visualization was done in R studio by corrplot package. *(*P* < 0.05), **(*P* < 0.01), ***(*P* < 0.001). B, ROC curve analysis was performed to evaluate the potential to predict five‐day survival, monocyte subsets showed significant predictive potential. AUC, area under curve; GMF, geometric mean of fluorescence; SOFA, sequential organ failure assessment

Differences in cytokine production between survivors and early deceased patients were analysed. The production of MCP‐1, IL‐6, IL‐8, IL‐10, and IL‐18 was significantly higher in early deceased patients than in those who survived to day 5 (Figure 2). Correlation analysis performed to determine the relationships between measured characteristics including T cells (Figure 3) are summarized in Figure 4A, values of correlations are presented in Figure S2. The reduced frequency of classical monocytes negatively correlated (*P* < 0.001) with the elevated frequency of intermediate and non‐classical monocytes. Reduced surface expression of activation markers—HLA‐DR and CD86 showed positive correlation related to each other in classical, intermediate and non‐classical monocytes. The decreased frequency of classical monocytes negatively correlated with the increased production of MCP‐1 (*P* < 0.01), IL‐6 (*P* < 0.01) and IL‐8 (*P* < 0.05). On the contrary, a positive correlation was observed between the elevated frequency of intermediate monocytes and the serum level of different cytokines—MCP‐1 (*P* < 0.001), IL‐6 (*P* < 0.01), IL‐8 (*P* < 0.05), IL‐10 (*P* < 0.01) and IL‐18 (*P* < 0.01). Significant negative correlations were also observed between the decreased CD86 expression levels in all monocyte subsets with increasing levels of majority of measured cytokines. Intermediate and classical monocyte subsets showed significant potential to predict risk of early death (AUC 0.894 for intermediate subset and AUC 0.767 for classical monocyte subset). Similarly to monocyte subsets, analysed cytokines may predict short‐term survival (Figure 4B).

### 3.4 T cell immunophenotype and correlation with cytokines levels

T cells were characterized by the presence of CD3, and subsequently, positivity on CD4 (T_h_) and CD8 (T_c_) was determined. The gating strategy is shown in Figure 3A.

Early deceased patients had significantly fewer CD4^+^ cells and significantly more CD8^+^ cells (*P* < 0.05) compared to patients who survived past day five (Figure 3B); the ratio of CD4^+^ and CD8^+^ populations was calculated (Figure 3D). No correlations between the frequency of CD4^+^ or CD8^+^ cells and the SOFA score were found (Figure 3D,E).

Correlation analysis was performed using individual parameters for immunophenotyping and cytokine production. The reduced frequency of CD4^+^ cells strongly negatively correlated with the increasing relative count of CD8^+^ cells (*P* < 0.001). The overall decreased frequency of CD4^+^ T cells was significantly associated with IL‐10 production (*P* < 0.05) (Figure 4, Figure S2.

## 4 DISCUSSION

Using data from a cohort of 41 patients admitted to the ICU with septic shock, we investigated whether the frequency of different monocyte subsets and their activation status assessed within 12 hours of admission, is associated with a fulminant course of septic shock. In order to identify the immune‐phenotype associated with fulminant course of septic shock, the studied cohort was retrospectively divided into two groups; one group contained patients with very short survival (within 5 days) and second was the group of patients, who survived this crucial 5‐day period. As we mainly focused to analyse phenotype of the dynamically changing monocytes subsets, the 12 hours time‐point of analysis provided an important advantage comparing to other reports focused to monocytes where authors used later sampling (1‐5 days after admission to ICU).^13^, ^14^, ^21^

The prompt risk stratification of patients with septic shock, is a key information for adjusting the care and later improvement of the therapeutics outcomes remain a key research focus.^5^, ^22^ Many different experimental strategies, including single cell expression analysis,^23^ have been used to improve our understanding of the immune response in the early phase of sepsis and to identify new therapeutic targets.^24^

Here we report shift in frequencies of classical and intermediate monocyte subset and strong negative correlation (*P* < 0.001) between these parameters. From this we speculate that the ‘inflammatory’ status of patients detected through frequency of intermediate monocytes reflects the septic shock severity, however the increased frequency of intermediate monocytes does not correlate with increasing SOFA score. Mukherjee et al and Liepelt et al also reported the similar changes in frequency of monocyte subsets of septic patients. They showed frequency of intermediate and also non‐classical monocytes, which was increased, whereas the frequency of classical monocyte subset was decreased in comparison to healthy controls.^21^, ^25^ Unlike published studies^21^, ^25^ comparing healthy volunteers, or critically ill non‐septic patients with septic patients, we used a more appropriate way to assess markers of septic shock prognosis dividing the cohort of septic shock patients to survivors and early deceased patients. We showed that shift in monocyte subsets can serve as predictive marker of early survival first five days of septic shock. The increased variability in monocyte population was also used as predictive marker of sepsis severity.^26^ Greco et al showed that intensified variability in monocyte population was originated by increased presence of intermediate and non‐classical monocytes.^27^

Interestingly, sepsis and septic shock affect not only monocyte subsets frequency, but also their activation status represented by surface expression of CD86 and HLA‐DR.^28^, ^29^ We observed significantly decreased surface expression of CD86 on classical monocytes and non‐significant decrease trend in intermediate and non‐classical monocytes. The changes in CD86 expression were also showed by Washburn et al, who did RNAseq of monocytes from septic patients and observed decreased expression levels of CD86 gene.^18^ We also focused on HLA‐DR surface expression levels. Our data showed a trend in decreased surface HLA‐DR expression in early deceased patients in comparison to D5+ survivors. HLA‐DR is well established predictive marker of sepsis seriousness,^30^, ^31^ moreover, reduced amounts of HLA‐DR result in functional impairment of antigen‐driven immunity, which can lead to increased susceptibility to secondary infections,^13^, ^32^, ^33^, ^34^, ^35^ nevertheless, its’ use never reached routine diagnostics. According to the dynamics of HLA‐DR and CD86 in sepsis, we determined the frequency of HLA‐DR^low,neg^ CD86^low,neg^ cells in all monocyte subsets and revealed a significantly increased frequency of HLA‐DR^low,neg^ CD86^low,neg^ in early deceased patients in comparison to patients, who survived day five. The population of HLA‐DR^low,neg^ CD86^low,neg^ cells across all monocyte subsets showed the correlations with various monocytic parameters and also with levels of produced cytokines. Furthermore, the increased frequency of HLA‐DR^low,neg^ CD86^low,neg^ showed significant correlation with severity of the septic shock.

Interestingly, it is well known that CD86, as a co‐stimulatory molecule, has a key role in supporting the development of T cells, therefore we also analysed basic changes in T cells. Considering our search for dynamically changing parameters among monocytes we also assessed some basic information from T cells immune‐phenotype, impaired T cells functions, exhaustion and apoptosis are hallmarks of sepsis.^18^ Although usually these changes are reported in later time‐points of septic shock and also can persist after patient recovery.^36^ Our data demonstrate the differences in CD4^+^ and CD8^+^ frequency occurred in both observed patient groups with septic shock at the time of their admission to ICU. The differences in CD4^+^ and CD8^+^ cell frequency to reduced amount CD4^+^ cells, probably indicates higher susceptibility to fulminant septic shock. Xia et al reported the decreased frequency of CD4^+^ cells and also CD4^+^/CD8^+^ ratio inclined to increased abundance of CD8^+^ cells, is associated with higher risk of development the postoperative sepsis in HIV‐infected patients.^37^ HIV‐infected patient usually have reduced numbers of CD4^+^ cells, because HIV triggers and kills CD4^+^ cells. Hotchkiss et al also observed, the depletion of CD4^+^ T cells induced by sepsis in non‐surviving patients.^17^ This phenomenon is highlighted by strong inverse correlation between the frequency of CD4^+^ and CD8^+^ T cells in our data. Furthermore, we also speculate, whether the significant changes in the surface levels of the key T cell co‐stimulatory molecule—CD86 observed on monocytes can eventually affect the pool of T cells. Others reported that decreased surface localization of CD86 can be associated with exhaustion and increased apoptosis of T cells.^19^

Sepsis and septic shock are associated with increased production of pro‐ and anti‐inflammatory mediators, also known as cytokine storm.^38^, ^39^ The promising results of earlier research on systemic markers of the inflammatory response, such as levels of cytokines originating from immune cells, have not been fully translated to clinical use eventually due to broad pleiotropic effects and diverse impact on immune cell populations in different timeframe of sepsis.

The changes in proportion from classical to intermediate and non‐classical monocyte subsets, are also associated with increased levels of produced pro‐inflammatory cytokines.^21^, ^40^ We correlated changes in monocyte subsets frequency together with secreted cytokines and evaluated their possible crosstalk. The reduced frequency of classical monocytes showed significant negative correlations with increased production of MCP‐1, IL‐6 and IL‐8, whereas elevated frequency of intermediate monocytes positively correlated with increased levels almost all measured cytokines. Thaler et al reported, that intermediate monocytes have increased IL‐6 and IL‐8 gene expression in septic patients.^40^ The detailed mechanism of changes in expression level is difficult to dissect as monocytes are also important producers of IL‐6 and IL‐8.

Similarly to monocyte subsets, reduced amounts of CD4^+^ T cells showed a negative correlation with elevated levels immunosuppressive cytokines IL‐10 and IL‐33 in our study. Increased expression of immunosuppression associated IL‐10 gene, was observed in T cells^18^ and also intermediate monocytes^25^ of septic patients. Nascimento et al observed that simultaneous activity of IL‐10 together with IL‐33 results in the long‐term immunosuppression by expanding T_reg_ cells in caecal ligation and puncture (CLP) mice model as well as in human suffering with sepsis.^41^

Our findings clearly highlight the possible predictive roles of monocyte subsets and their activation markers in short‐term septic shock survival. Furthermore, we suggest an important crosstalk of changes in monocytes with changes in T cell compartment, possibly explained through the cytokine levels changes or CD86 expression. However, validation of our data will be required as our study had several limitations including size (41 patients) recruited from a single centre; the average age of patients was 71.3 years, which limits how our findings can be generalized to other age groups.

Even though we analysed data from cohort with a very broad clinical sources of septic shock including different types of bacterial agents, our results demonstrate that monocyte subset frequency can serve as predictive marker of septic shock survival independent on stimuli, which induced the septic shock. Further analysis in a larger cohort will allow us to determine whether the observed differences in monocyte subsets can be used to predict patient outcome and be used to risk stratify patients and detect those with excessive inflammatory response who may profit from immunomodulatory therapy.

**FIGURE 5:**
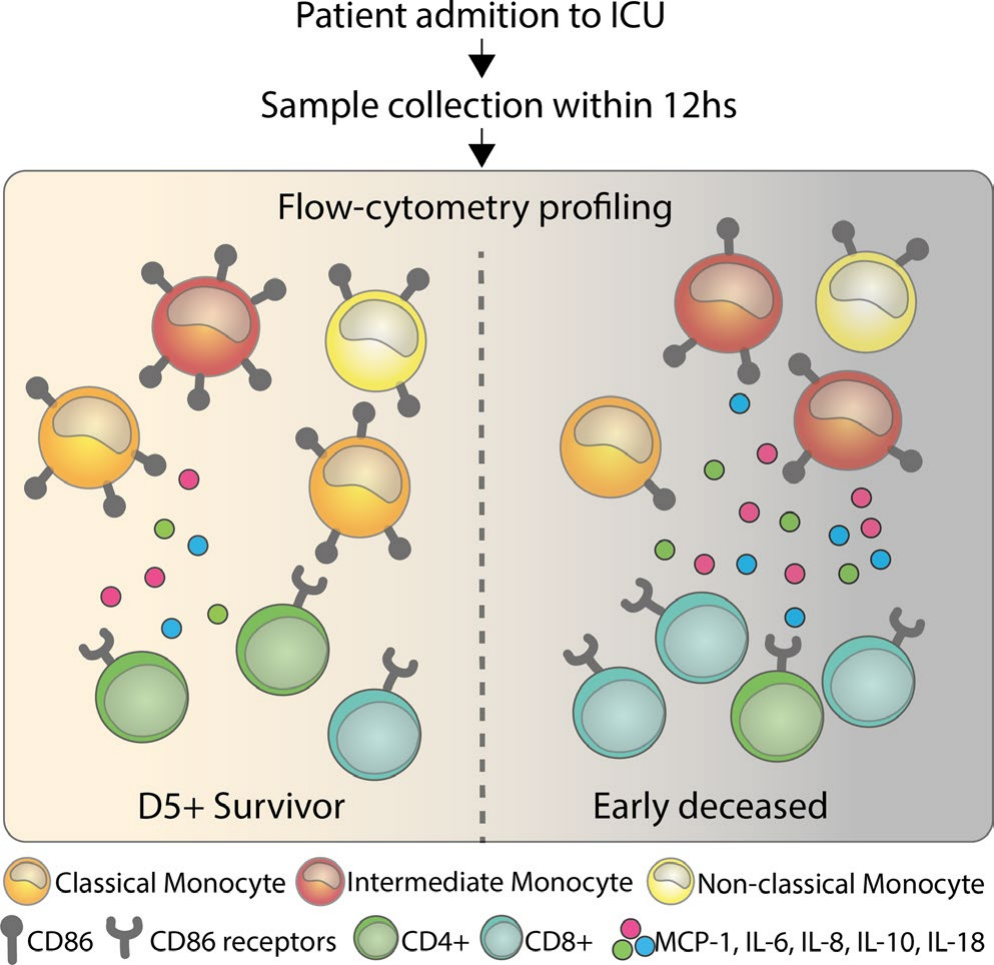
The changes in monocyte subset frequency, reduced status of their activation and increased cytokine production can serve as predictive marker of septic shock prognosis

Altogether, we demonstrated that proportional changes in monocyte subsets and their correlation with increased cytokine production and decreased status of activation of these cells, can serve as predictive marker of septic shock prognosis (Figure 5). Future research should deeply focus on the dynamics of monocytes subsets in progression of septic shock. We have identified markers that have the potential to be used in prognostic tests; the sample may be taken and analysed at the time of admission, and parameters correlating with survival quantified within hours of sample collection. This approach may help to apply more tailored immunotherapy of patients in septic shock.

## CONFLICT OF INTEREST

Authors have no conflicting interests.

## AUTHOR CONTRIBUTIONS

**Marcela Hortová Kohoutková:** Conceptualization (equal); Data curation (lead); Formal analysis (equal); Visualization (lead); Writing‐original draft (equal); Writing‐review & editing (equal). **Petra Lázničková:** Data curation (equal); Formal analysis (equal); Writing‐review & editing (equal). **Kamila Bendíčková:** Conceptualization (equal); Data curation (equal); Formal analysis (equal); Writing‐review & editing (equal). **Marco De Zuani:** Data curation (equal); Writing‐review & editing (equal). **Ivana Andrejčinová:** Data curation (equal); Writing‐review & editing (equal). **Veronika Tomášková:** Data curation (equal). **Pavel Suk:** Writing‐review & editing (equal). **Vladimír Šrámek:** Data curation (equal); Supervision (equal); Writing‐review & editing (equal). **Martin Helán:** Conceptualization (equal); Data curation (equal); Investigation (equal); Writing‐review & editing (equal). **Jan Fric:** Conceptualization (equal); Funding acquisition (lead); Investigation (equal); Supervision (lead); Writing‐original draft (equal); Writing‐review & editing (equal).

## Supporting information

Fig S1‐S2

## Data Availability

Data availability is upon the reasonable request from the corresponding author. The data are not publicly available due to privacy or ethical restrictions.
