# Supplementary material for: Differences in monocyte subsets are associated with short‐term survival in patients with septic shock
Source: J Cell Mol Med. 2020 Sep 19;24(21):12504–12. doi: 10.1111/jcmm.15791 (PMC7686971; doi:10.1111/jcmm.15791)
Supplement: Supplementary file 1 — Fig S1‐S2 [file JCMM-24-12504-s001.pdf]

Supplemental Information

Differences in monocyte subsets are associated with short-term survival in patients with septic shock

Marcela Hortová-Kohoutková[1], Petra Lázničková[1,2], Kamila Bendíčková[1], Marco De Zuani[1], Ivana Andrejčinová[1,2], Veronika Tomášková[1,3], Pavel Suk[1,3], Vladimír Šrámek[3], Martin Helán[1,3], and Jan Frič[1,4*]

[1] International Clinical Research Center, St. Anne's University Hospital Brno, Brno.
[2] Department of Biology, Faculty of Medicine, Masaryk University, Brno, Czech Republic
[3] Department of Anesthesiology and Intensive Care, Faculty of Medicine, Masaryk University, Brno, Czech Republic.
[4] Institute of Hematology and Blood Transfusion, Prague, Czech Republic

* Corresponding author: e-mail: jan.fric@fnusa.cz

Figure S1, related to Table 1

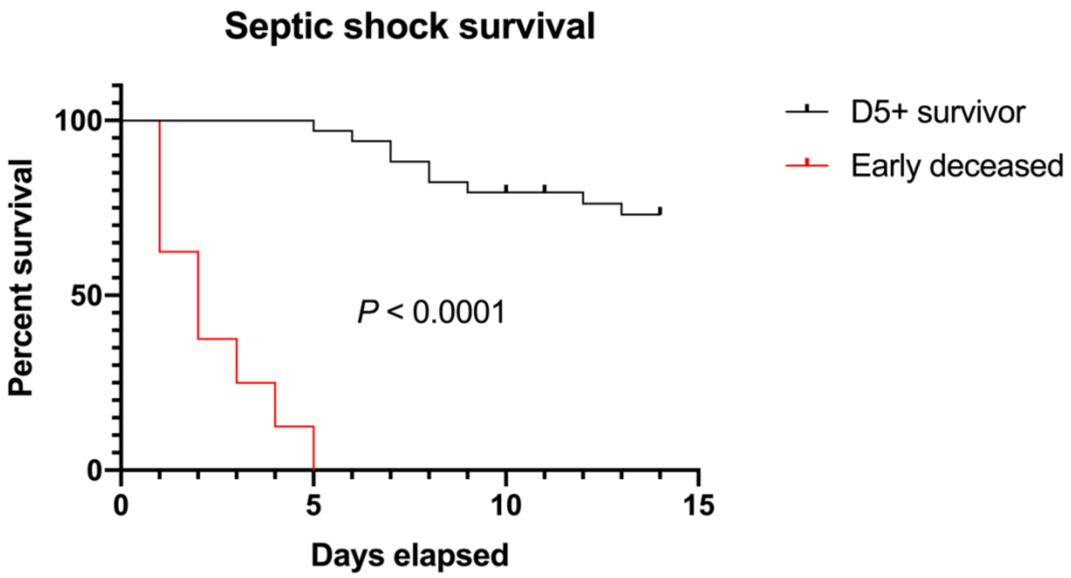

Figure S1 | Comparison of survivor curves of D5+ survivors and Early deceased patients with septic shock.

Figure S2, related to Figure 4

				Classical monocytes				Intermediate monocytes				Non-classical monocytes				T cells			Cytokines & chemokines					
		SOFA	Lactate	frequency (%)	HLA-DR+ (GMF)	CD86+ (GMF)	HLA-DRlo/- CD86lo/- (%)	frequency (%)	HLA-DR+ (GMF)	CD86+ (GMF)	HLA-DRlo/- CD86lo/-	frequency (%)	HLA-DR+ (GMF)	CD86+ (GMF)	HLA-DRlo/- CD86lo/-	CD4+	CD8+	CD4+/CD8+ ratio	MCP-1	IL-6	IL-8	IL-10	IL-18	IL-33
	SOFA		0.234	-0.205	-0.281	-0.211	0.348	0.235	-0.207	-0.297	0.256	-0.082	-0.165	-0.166	0.334	0.161	-0.175	0.035	0.272	0.244	0.255	0.189	0.225	0.163
	Lactate	0.234		-0.424	-0.307	-0.337	0.508	0.507	-0.311	-0.460	0.477	-0.173	-0.291	-0.384	0.371	-0.135	0.052	-0.106	0.697	0.662	0.603	0.706	0.176	0.296
Classical	frequency (%)	-0.205	-0.424		-0.074	0.048	-0.309	-0.733	-0.184	0.074	-0.295	-0.590	-0.148	0.017	-0.199	0.031	-0.044	0.067	-0.485	-0.407	-0.384	-0.310	-0.276	0.009
	HLA-DR+ (GMF)	-0.281	-0.307	-0.074		0.715	-0.478	-0.144	0.791	0.730	-0.270	0.167	0.688	0.403	-0.113	-0.169	0.288	-0.222	-0.242	-0.192	-0.160	-0.247	-0.281	-0.278
	CD86+ (GMF)	-0.211	-0.337	0.048	0.715		-0.743	-0.326	0.725	0.736	-0.370	0.069	0.727	0.675	-0.374	0.021	0.101	-0.099	-0.379	-0.334	-0.303	-0.324	-0.381	-0.307
	HLA-DR lo/- CD86lo/- (%)	0.348	0.508	-0.309	-0.478	-0.743		0.487	-0.368	-0.689	0.582	-0.096	-0.338	-0.572	0.549	-0.130	-0.044	-0.037	0.554	0.549	0.518	0.485	0.522	0.333
Intermediat	frequency (%)	0.235	0.507	-0.733	-0.144	-0.326	0.487		-0.159	-0.241	0.254	0.229	-0.158	-0.131	0.124	-0.050	0.079	-0.152	0.569	0.439	0.377	0.467	0.471	0.073
	HLA-DR+ (GMF)	-0.207	-0.311	-0.184	0.791	0.725	-0.368	-0.159		0.546	-0.242	0.310	0.964	0.551	-0.186	-0.210	0.305	-0.233	-0.240	-0.150	-0.121	-0.211	-0.247	-0.202
	CD86+ (GMF)	-0.297	-0.460	0.074	0.730	0.736	-0.689	-0.241	0.546		-0.546	0.192	0.465	0.630	-0.358	0.044	0.128	-0.119	-0.413	-0.416	-0.384	-0.363	-0.341	-0.336
	HLA-DR lo/- CD86lo/- (%)	0.256	0.477	-0.295	-0.270	-0.370	0.582	0.254	-0.242	-0.546		-0.114	-0.207	-0.439	0.620	0.002	-0.047	-0.047	0.603	0.527	0.511	0.230	0.355	0.139
Non-classical	frequency (%)	-0.082	-0.173	-0.590	0.167	0.069	-0.096	0.229	0.310	0.192	-0.114		0.246	0.228	-0.105	-0.029	0.059	-0.012	-0.076	-0.062	-0.055	-0.121	-0.010	-0.204
	HLA-DR+ (GMF)	-0.165	-0.291	-0.148	0.688	0.727	-0.338	-0.158	0.964	0.465	-0.207	0.246		0.631	-0.259	-0.161	0.247	-0.196	-0.208	-0.145	-0.125	-0.187	-0.215	-0.188
	CD86+ (GMF)	-0.166	-0.384	0.017	0.403	0.675	-0.572	-0.131	0.551	0.630	-0.439	0.228	0.631		-0.726	0.150	0.042	0.003	-0.322	-0.376	-0.393	-0.252	-0.062	-0.348
	HLA-DR lo/- CD86lo/- (%)	0.334	0.371	-0.199	-0.113	-0.374	0.549	0.124	-0.186	-0.358	0.620	-0.105	-0.259	-0.726		-0.103	0.000	-0.104	0.351	0.460	0.505	0.176	0.055	0.322
T cells	CD4+	0.161	-0.135	0.031	-0.169	0.021	-0.130	-0.050	-0.210	0.044	0.002	-0.029	-0.161	0.150	-0.103		-0.882	0.806	-0.060	-0.257	-0.194	-0.375	0.157	-0.187
	CD8+	-0.175	0.052	-0.044	0.288	0.101	-0.044	0.079	0.305	0.128	-0.047	0.059	0.247	0.042	0.000	-0.882		-0.873	-0.008	0.173	0.116	0.302	-0.136	0.147
	CD4+/CD8+ ratio	0.035	-0.106	0.067	-0.222	-0.099	-0.037	-0.152	-0.233	-0.119	-0.047	-0.012	-0.196	0.003	-0.104	0.806	-0.873		-0.054	-0.223	-0.176	-0.259	0.179	-0.119
Cytokines & chemikines	MCP-1	0.272	0.697	-0.485	-0.242	-0.379	0.554	0.569	-0.240	-0.413	0.603	-0.076	-0.208	-0.322	0.351	-0.060	-0.008	-0.054		0.819	0.767	0.687	0.304	0.114
	IL-6	0.244	0.662	-0.407	-0.192	-0.334	0.549	0.439	-0.150	-0.416	0.527	-0.062	-0.145	-0.376	0.460	-0.257	0.173	-0.223	0.819		0.969	0.753	0.113	0.239
	IL-8	0.255	0.603	-0.384	-0.160	-0.303	0.518	0.377	-0.121	-0.384	0.511	-0.055	-0.125	-0.393	0.505	-0.194	0.116	-0.176	0.767	0.969		0.622	0.049	0.171
	IL-10	0.189	0.706	-0.310	-0.247	-0.324	0.485	0.467	-0.211	-0.363	0.230	-0.121	-0.187	-0.252	0.176	-0.375	0.302	-0.259	0.687	0.753	0.622		0.221	0.340
	IL-18	0.225	0.176	-0.276	-0.281	-0.381	0.522	0.471	-0.247	-0.341	0.355	-0.010	-0.215	-0.062	0.055	0.157	-0.136	0.179	0.304	0.113	0.049	0.221		0.011
	IL-33	0.163	0.296	0.009	-0.278	-0.307	0.333	0.073	-0.202	-0.336	0.139	-0.204	-0.188	-0.348	0.322	-0.187	0.147	-0.119	0.114	0.239	0.171	0.340	0.011	

Figure S2 | Analysis of correlations between monocyte, T cell parameters with produced cytokines.

Measured parameters of monocyte and T cells, together with secreted cytokines were analyzed and correlation analysis was performed using Spearman's rank correlation coefficient.
